# Theoretical study on thermal decomposition mechanism of 1-nitroso-2-naphthol

**DOI:** 10.1038/s41598-022-24638-z

**Published:** 2022-11-21

**Authors:** Xiaohua Fan, Yixiu Gan, Miaowen Tan, Wenhe Wang

**Affiliations:** 1 School of Safety Engineering, Chongqing University of Science and Technology, Chongqing, 401331 China; 2 Institute of Chemical Technology, Dalian University of Technology, Dalian, 116024 China

**Keywords:** Chemical safety, Engineering

## Abstract

1-nitroso-2-naphthol has thermal instability of thermal decomposition, spontaneous combustion and even explosion. Its thermal decomposition characteristics were tested by synchronous thermal analyzer (TGA/DSC); The activation energy of the thermal decomposition process was calculated by Kissinger method; The infrared absorption characteristic spectra of the gas products produced in the thermal decomposition process were measured by TGA/DSC-FTIR, and the thermal decomposition reaction process was speculated. The results show that the initial temperature (*T*_onset_) of TGA exothermic decomposition of 1-nitroso-2 naphthol is between 129.01 and 155.69 °C, and the faster the heating rate(β), the higher the *T*_onset_, but the faster the thermal decomposition rate, the greater the heat release and the worse the thermal stability. The activation energy (E) of the thermal decomposition process is 83.323 kJ/mol calculated by Kissinger method. The dynamic test results of TGA/DSC-FTIR show that the main reaction of 1-nitroso-2 naphthol during heating is intermolecular dehydration to form ether, and the secondary reaction is decomposition into aliphatic nitro compounds, carbonyl compounds and amines. Sodium hydroxide will reduce the thermal stability of 1-nitroso-2 naphthol. After adding sodium hydroxide, the thermal decomposition process of 1-nitroso-2 naphthol has changed. The main reaction is that 1-nitroso-2-naphthol reacts with sodium hydroxide to produce sodium nitrophenol, which is further decomposed into aliphatic nitro compounds. The research results have guiding significance for finding the reasonable conditions and temperature of 1-nitroso-2 naphthol during storage and transportation.

## Introduction

1-nitroso-2-naphthol can be widely used in chemical and pharmaceutical fields as chelating agent and chromogenic agent. However, as a nitro compound, 1-nitroso-2-naphthol has certain thermal instability and can undergo exothermic decomposition after heating. Especially when it encounters acid or alkali, it will spontaneously ignite immediately, so it has certain thermal hazard.

At present, in the field of material thermal hazard research, the research objects are mainly energetic materials^1^, including organic peroxides and nitro compounds, such as cumene hydroperoxide^2^, benzoyl peroxide^3^, ammonium nitrate^4^, guanidine nitrate^5^, etc. The risk of thermal decomposition of these substances were be studied. The effects of acid^6^, alkali^7^, metal ion^8^, organic matter^9^ on the thermal decomposition process of these substances were be researched, etc.

The research method of material thermal safety mainly uses DSC^10^, TGA^11^, arc^12^, RC1^13^, C80^14^,C600^15^, VSPII^16^ and other thermal analysis instruments to test the thermal decomposition process of materials, and uses Kissinger method^17^, Flynn wall Ozawa method^18^, Starink method^19^ and so on, to calculate the activation energy of material thermal decomposition reaction and thermodynamic parameters, so as to evaluate the thermal hazard risk of the substance. For example, Xia, et al.^20^ and others studied the thermal decomposition characteristics and thermal risk of three anthraquinone hazardous waste by differential scanning calorimetry (DSC), calculated the kinetic characteristics of the decomposition process by Friedman method, and the effect of the coupling of phase transition and decomposition on the thermal risk of materials was studied. Yabei Xu et al.^21^ studied the Autocatalytic Decomposition Characteristics and thermal decomposition of benzoyl peroxide by differential scanning calorimetry (DSC), and calculated the kinetic parameters of the decomposition process by Kissinger method; Suranee et al.^22^ Evaluated the thermal hazard and reactivity of hydrogen peroxide with a mass concentration of 35% by DSC. It is found that the calculated activation energy is 70.03 kJ/mol, and the adiabatic temperature rise at heating rates of 2, 4, 8 °C/min is 236.5, 159.2, 217.5 k.

So far, the research on 1-nitroso-2-naphthol mainly focuses on the determination of cobalt, palladium, copper and iron^23^, and the research on its thermal stability has not been reported. In this paper, the thermal decomposition process of 1-nitroso-2-naphthol was analyzed and evaluated by TGA/DSC-FTIR. The effects of heating rate and impurities on the thermal decomposition of 1-nitroso-2-naphthol were studied by TGA/DSC. The activation energy of the thermal decomposition process was calculated by Kissinger method. The infrared absorption spectra of the gas products in the decomposition process were measured by TGA/DSC-FTIR, the group characteristics of the gas decomposition products were analyzed, and the reaction process of the decomposition process was speculated. The research results of this paper have certain reference significance for the storage and transportation safety of 1-nitroso-2 naphthol.

## Experimental

### Instruments and reagents

Main reagents and apparatuses used in the experiment are shown in Tables 1 and 2 respectively.

**Table 1:** List of experimental reagents.

Reagent	Molecular formula	Specifications	Manufacturer
1-nitroso-2 naphthol	C_10_H_7_NO_2_	Chemically pure, solid	*Shanghai McLean* Electronics Co
Sodium hydroxide (flake)	NaOH	Analytical purity, solid	Chron Chemicals

**Table 2:** List of experimental instruments.

Instrument	Model	Manufacturer
Electronic Ealance	3002	Hangzhou Youheng Weighting Equipment Cp.,Ltd
Fourier infrared spectrometer (FTIR)	Nicolet iS10	ThermoFisher Scientific
Differential scanning calorimeter	TGA/DSC 3 +	METTLER TOLED

### Differential scanning calorimetry experiments

TGA/DSC 3 + ecalorimeter manufactured by Mettler Corporation in Switzerland was used to obtain the mass change, the thermal endothermic and exothermic characteristics and the initial thermodynamic parameters of the material in the heating environment. TGA/DSC involves setting the test method, testing the blank curve, weighing a small amount of 1-nitroso-2-naphthol sample(5–10 mg), placing the sample in a crucible and subsequently on the thermal detector along with an empty crucible (a reference). Then start testing things. The Crucible material is alumina, Method gas is N_2_ with a flow rate of 5 ml/min, Temperature range is 30–300 °C, heating rate (β) is 5–25 °C/min.

### TGA/DSC-FTIR experiments

Fourier transform infrared spectroscopy (FTIR) is based on the principle that molecules interact with electromagnetic radiation in the near infrared (12,500 ~ 4000 cm^−1^), mid infrared (4000–200 cm^−1^) and far infrared (200 ~ 12.5 cm^−1^) spectral regions. When infrared radiation passes through a sample, the sample will absorb energy of a certain frequency according to the structural characteristics of different molecules, causing the molecules or different parts of the molecules (functional groups) to vibrate at these frequencies, Get the structure information of the functional groups of the molecule.

Connecting the thermal analyzer and the infrared spectrometer in series through a heatable transmission pipeline is called thermal infrared combined technology (TGA/DSC-FTIR). This method uses purge gas (usually nitrogen or air) to transfer the fugitive products generated by the TGA/DSC during heating to the gas pool in the optical path of FTIR through a high temperature (usually 200 °C–350 °C) metal pipe, It is a technique to analyze and judge the component structure of escaping gas through the detector of infrared spectrometer (MCT detector). During the experiment, with the temperature change of TGA/DSC, while the change of the mass and heat flow of the sample to be measured with the temperature, the infrared spectrometer measures the functional group information of the gas products overflowed at different temperatures. Therefore, this method can be used to infer the process of thermal decomposition.

In this paper, the purging gas is nitrogen, its flow rate is 50 ml/min, the pipeline transmission temperature is 260℃, and the resolution is 4.

## Results and discussion

### TGA/DSC thermal decomposition characteristics of 1-nitroso-2-naphthol

**Figure 1:**
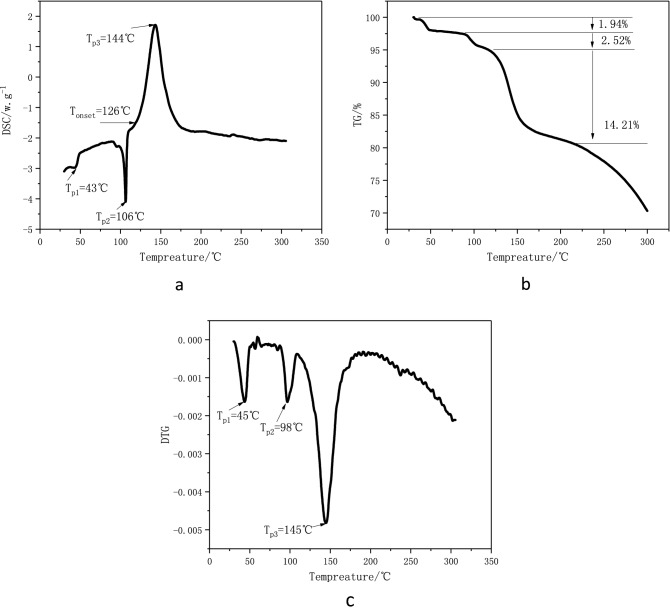
DSC curve (**a**)、TG curve (**b**) and DTG curve (**c**) of 1-nitroso-2-naphthol at 5 °C/min.

The thermal decomposition data of 1-nitroso-2-naphthol obtained by TGA/DSC at the heating rate of 5 °C/min are shown in Fig. 1. As can be seen from Fig. 1a, 1-nitroso-2-naphthol has three heat flow peaks during heating. The first two are endothermic peaks. The first endothermic peak is relatively small, and the peak temperature (*T*_p1_) is about 43 °C, which may be formed by the evaporation of water in 1-nitroso-2-naphthol. The second endothermic peak is relatively obvious, and the peak temperature (*T*_p2_) is about 106 °C, indicating that part of 1-nitroso-2-naphthol changes its phase state through endothermic. The third peak is an obvious exothermic peak, and initial exothermic temperature (*T*_onset_) is about 126 °C, and the peak temperature (*T*_p3_) is about 144 °C, indicating that 1-nitroso-2-naphthol has undergone thermal decomposition and released a lot of heat.

These three heat flow peaks of DSC correspond to the three weight loss steps of TGA curve in Fig. 1b and the three weight loss peaks of DTG curve in Fig. 1c, that is, the peak temperature (TP1) of the first weightlessness is about 45 °C, the weight loss is about 1.94%, and the peak temperature (TP2) of the second weightlessness is about 98 °C, the weight loss rate is about 2.52%. Both weightlessness may be caused by the volatilization of the adsorbed water in the sample. The peak temperature (*T*_p3_) of the third weight loss is about 145 °C, and the weight loss was about 14.21%, corresponding to the thermal decomposition reaction of the sample.

### Effect of sodium hydroxide on thermal stability of 1-nitroso-2-naphthol

It is reported that the existence of impurities has a certain impact on the thermal stability of substances ^24,25^. It can be inferred from the production process of 1-nitroso-2-naphthol that 1-nitroso-2-naphthol may be mixed with unreacted sodium hydroxide in the production process. Therefore, the effect of sodium hydroxide on the thermal decomposition behavior of 1-nitroso-2-naphthol was studied by TGA / DSC-FTIR.

**Figure 2:**
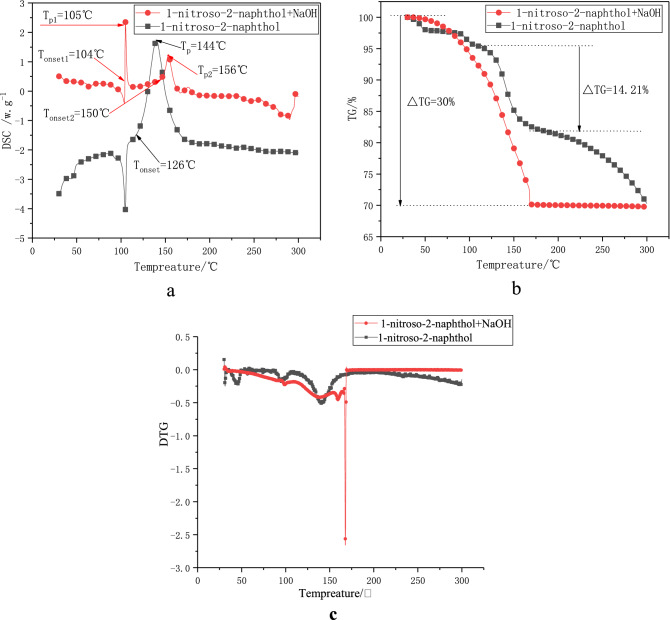
DSC curve (**a**) 、TG curve (**b**) and DTG curve (**c**) of 1-nitroso-2-naphthol before and after adding sodium hydroxide.

The content of sodium hydroxide is 5%, and the test method is the same as TGA/DSC. The test results are shown in Fig. 2.

According to Fig. 2a, there are two significant exothermic peaks in the DSC curve of 1-nitroso-2-naphthol added with sodium hydroxide. The initial exothermic temperature (*T*_onset1_) of the first exothermic peak is 104 °C and the peak temperature(T_p1_) is 105 °C, which were lower than that of pure 1-nitroso-2-naphthol. It shows that 1-nitroso-2-naphthol is more prone to exothermic decomposition after adding sodium hydroxide at room temperature. Compared with the DSC curve of pure 1-nitroso-2-naphthol, the DSC curve of 1-nitroso-2-naphthol with sodium hydroxide has a second exothermic peak. The initial exothermic temperature of the second exothermic peak (*T*_onset2_) is 150 °C and the peak temperature (*T*_p2_) is 156 °C, indicating that sodium hydroxide will promote the secondary thermal decomposition of 1-nitroso-2-naphthol in the later stage of the reaction and release a certain amount of heat.

The TG diagram in Fig. 2b and DTG diagram in Fig. 2c can also confirm that after the addition of sodium hydroxide, the thermal weight loss of 1-nitroso-2-naphthol increases and the rate of thermal weight loss accelerates. This shows that sodium hydroxide will reduce the thermal stability of 1-nitroso-2-naphthol.

### Effect of heating rate (β) on thermal decomposition of 1-nitroso-2-naphthol

**Figure 3:**
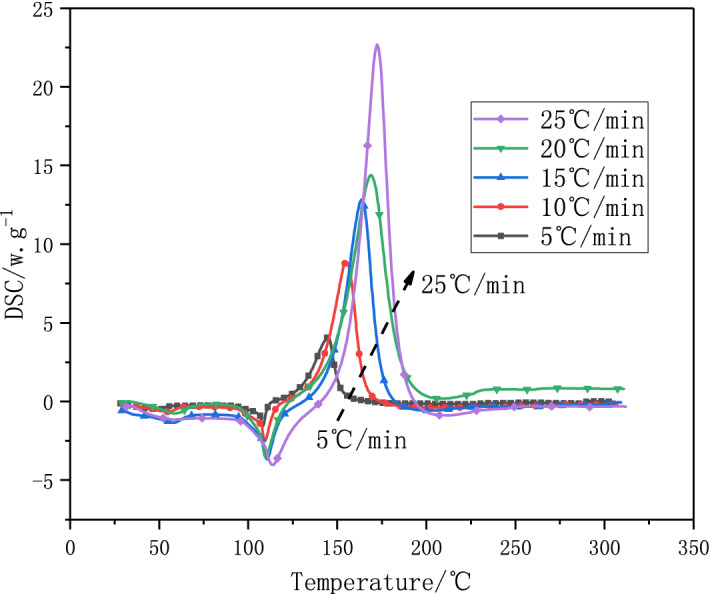
DSC curves of 1-nitroso-2-naphthol at different heating rates.

**Figure 4:**
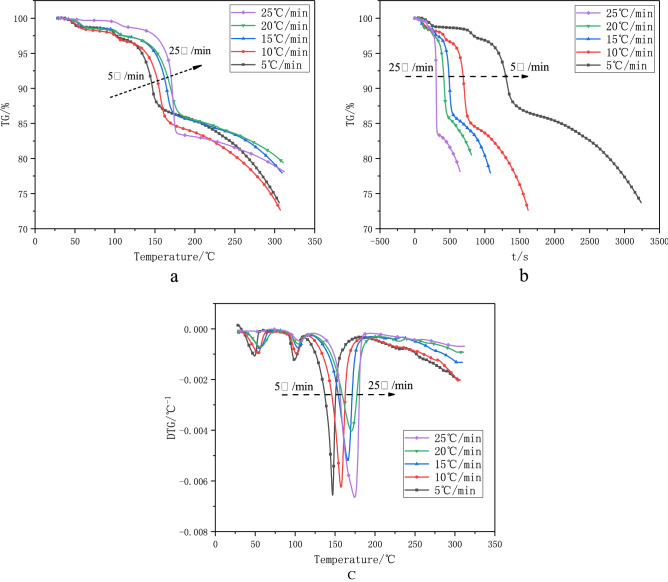
TGA -temperature diagram (**a**) 、TGA-time diagram (**b**) and DTG- temperature diagram (**c**) of 1-nitroso-2-naphthol at different heating rates.

In order to study the effect of different heating rates on the thermal decomposition of 1-nitroso-2-naphthol. Five different heating rates (5, 10, 15, 20, 25 °C/min) were used to study the thermal decomposition of 1-nitroso-2-naphthol. The results are shown in Figs. 3 and 4. It can be seen from Fig. 3 that with the increase of heating rate (β), the peak temperature (*T*_p_) and initial exothermic temperature (*T*_onset_) tend to move to the right, and the shape of exothermic peak of DSC heat flow curve becomes more and more sharp. This shows that the heating rate (β) will affect the thermal decomposition of 1-nitroso-2-naphthol. With the increase of heating rate (β), the initial decomposition temperature (*T*_onset_) of 1-nitroso-2-naphthol increases. This is because the heating rate is too fast, the substances are heated unevenly, and some substances have no time to decompose, so the thermal decomposition temperature is postponed. However, the faster the heating rate, the larger the area of heat release peak, the greater the heat release, the faster the heat release rate and the worse the thermal stability. The test result data are shown in Table 3.

**Table 3:** DSC exothermic data of 1-nitroso-2-naphthol.

Sample	Heating rate(β)/ °C min^−1^	$T_{onset}$/°C	$T_{p}$/°C	ΔH(J/g)
1-nitroso-2-naphthol	5	126.01	144.57	705.43
	10	139.93	155.01	766.72
	15	146.47	163.76	863.55
	20	147.83	169.07	885.63
	25	155.69	172.33	892.56

The thermogravimetric (TG) curves at different heating rates are shown in Fig. 4.

The thermogravimetric diagram in Fig. 4 also confirms the phenomenon that the initial decomposition temperature (*T*_onset_) of 1-nitroso-2-naphthol increases with the increase of heating rate(β) (Fig. 4a and c). With the increase of heating rate (β), the TGA curve of 1-nitroso-2-naphthol moves to the right. However, it can also be seen from TGA-time diagram (Fig. 4b) that with the increase of heating rate (β), the thermal decomposition weight loss time of 1-nitroso-2-naphthol becomes shorter, the weight loss rate increases and the thermal safety becomes worse.

### Analysis of thermal decomposition kinetic parameters of 1-nitroso-2-naphthol

According to the DTG peak data (*T*_p,i_) at different heating rates (β) in the previous paper, the thermal decomposition kinetic parameters of 1-nitroso-2-naphthol were calculated by Kissinger method^16,26^. Kissinger's formula is as follows:

1 $$ \ln \left( {\frac{\beta }{{T_{p,i}^{2} }}} \right) = \ln \left( { - \frac{AR}{E}f^{\prime}\left( {\alpha_{p} } \right)} \right) - \frac{E}{{RT_{p,i} }}\quad (i = 1,2,...,{\text{n}}) $$

Kissinger's method points out that $f^{\prime } \left( {\alpha_{p} } \right)$ will not change with the change of $\beta$, and the calculation of kinetic mechanism function can be approximately 1. Therefore, according to the linear relationship between $\ln \left( {\frac{\beta }{{T_{p,i}^{2} }}} \right)$ and $\frac{1}{{T_{p,i} }}$, this method performs linear fitting analysis on the peak temperature ($T_{p,i}$) at different heating rates(β_i_), and uses the linear slope to solve the reaction activation energy(E).

**Figure 5:**
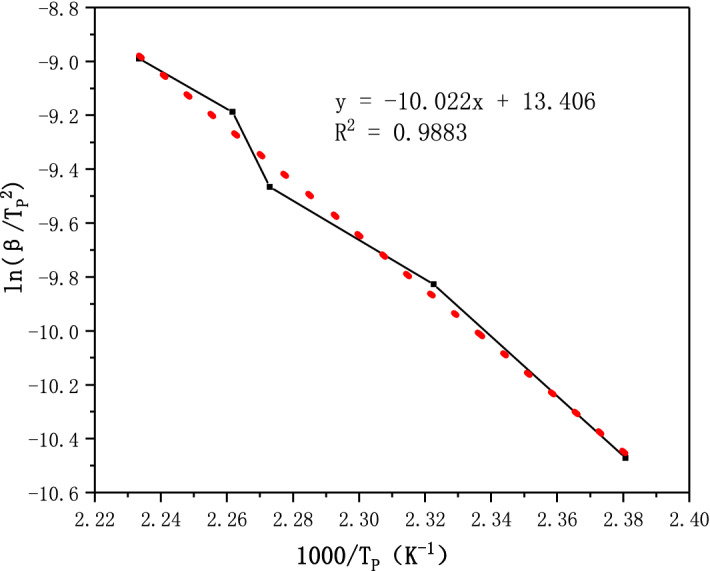
Fitting Kissinger method results of 1-nitroso-2-naphthol.

According to the decomposition exothermic peak($T_{p,i}$) in DTG curves with heating rates (β_i_) of 5, 10, 15, 20 and 25 °C /min, The correlation diagram is drawn with $\ln \left( {\frac{\beta }{{T_{p,i}^{2} }}} \right)$ as the ordinate and $\frac{1000}{{T_{{}} }}$ as the abscissa. The five peak data points are linearly fitted. The calculation results are shown in Fig. 5.

It can be seen from the fitting results in Fig. 5 that the linear correlation coefficient (R^2^) is 0.9883 and the correlation is good. From the slope E/R = 10.022, it can be calculated that the activation energy E of 1-nitroso-2-naphthol thermal decomposition reaction is 83.323 kJ/mol, and the activation energy is low, indicating that the thermal decomposition reaction of 1-nitroso-2-naphthol is easy and has great thermal risk.

### Mechanism analysis of thermal decomposition reaction of 1-nitroso-2 naphthol

TGA–DSC-FTIR can dynamically and real-time scan the infrared spectrum information of the gas products released by the decomposition of 1-nitroso-2-naphthol during TGA test. Then, according to the infrared absorption standard spectrum, the composition of gas products produced in each stage of decomposition is analyzed, and the thermal decomposition process of 1-nitroso-2-naphthol is speculated.

**Figure 6:**
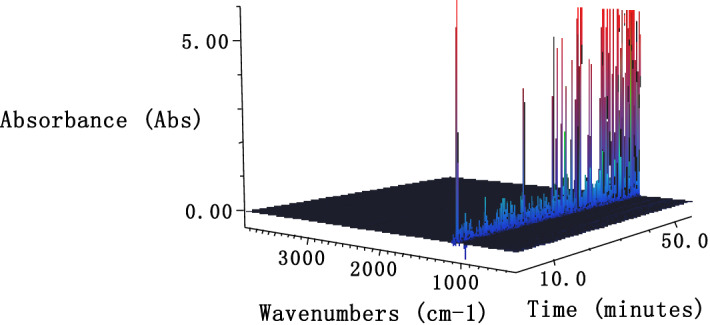
Series 3-D FTIR of gas products from thermal decomposition of 1-nitroso-2-naphthol.

**Figure 7:**
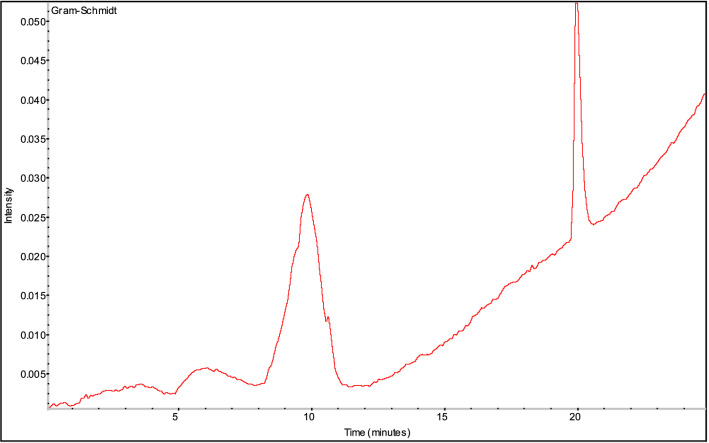
Gram-Schmidt of gas products from thermal decomposition of 1-nitroso-2-naphthol.

The dynamic three-dimensional infrared spectrum and Gram-Schmidt information of the gas products decomposed by 1-nitroso-2-naphthol during heating is shown in Figs. 6 and 7, respectively.

As can be seen from Fig. 6, in the whole thermal decomposition process, there is always absorption near 1100 cm^−1^, especially in the later stage of the reaction, the absorption at 1100 cm^−1^ is stronger. Compared with the infrared standard spectrum, 1100 cm^−1^ is the antisymmetric stretching vibration peak of C–O–C. It is reported that as long as it is an ether compound, this peak is often the strongest peak in the spectrum, which is more characteristic. Therefore, it can be inferred that there may be dehydration reaction between 1-nitroso-2-naphthol molecules to form ether.

**Figure 8:**
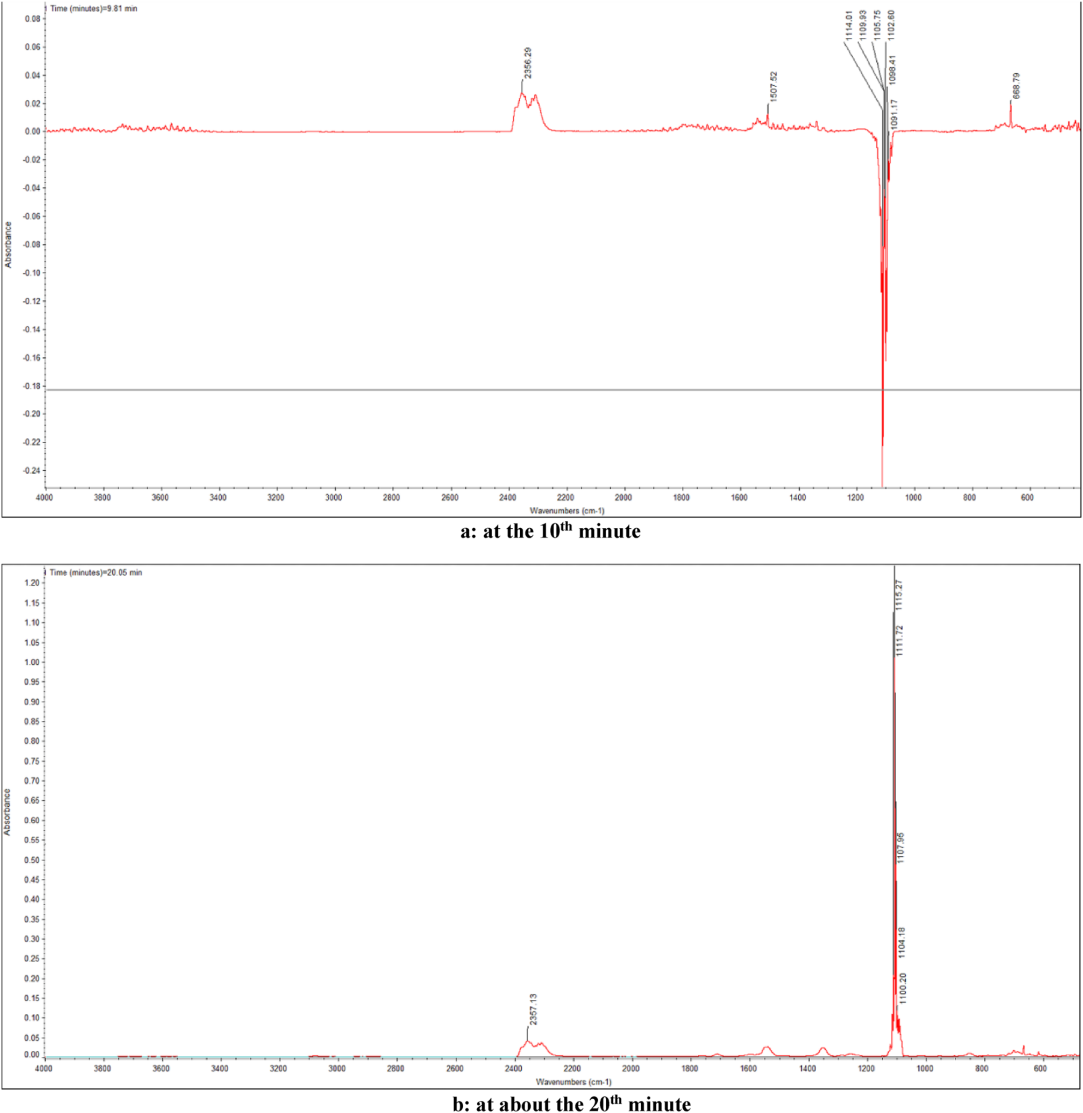
FTIR of gas products from thermal decomposition of 1-nitroso-2-naphthol at the 10^th^ minute and the 20th minute.

It can be seen from Fig. 7 that the strong and dense absorption is at about the 10^th^ minute and the 20th minute, so the infrared absorption spectrum at that time is listed and analyzed separately, as shown in Fig. 8.

As can be seen from Fig. 8, in addition to the strong absorption peak of antisymmetric stretching vibration of ether bond C–O–C near 1100 cm^−1^, there is also a certain intensity of infrared absorption near 668 cm^-1^ and 2356 cm^−1^. It is found that 668 cm^−1^ and 2356 cm^−1^ correspond to in-plane and out of plane bending vibrations and asymmetric stretching peaks of CO_2_, respectively.

It can be inferred from the positions of these absorption peaks in Fig. 8 that 1-nitroso-2-naphthol mainly undergoes intermolecular dehydration reaction to form ether during heating, and decomposes to release a small amount of carbon dioxide.

**Figure 9:**
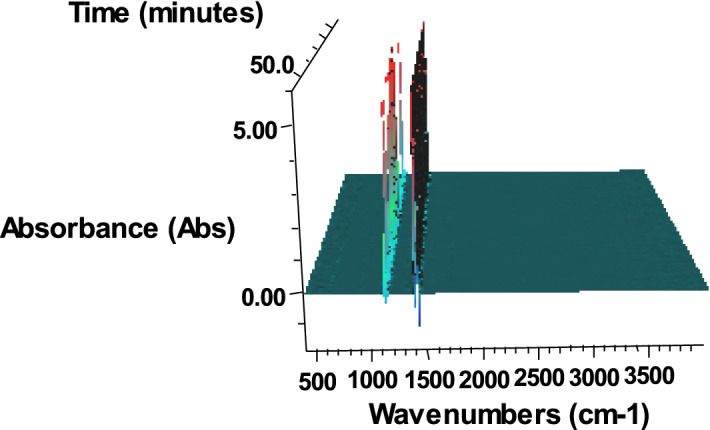
Series 3-D FTIR of gas products from thermal decomposition of 1-nitroso-2-naphthol + 5%NaOH.

**Figure 10:**
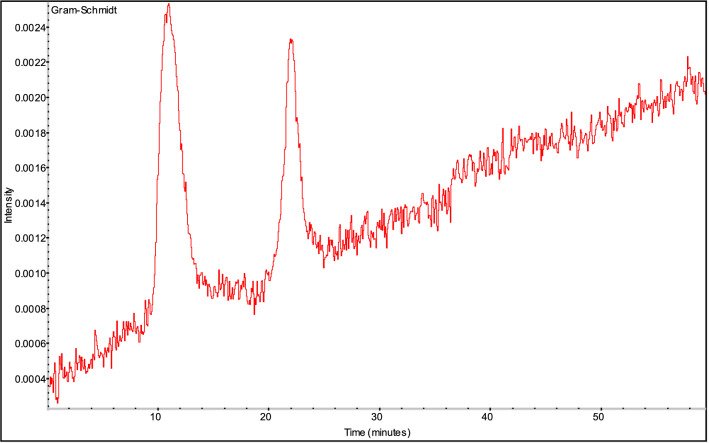
Gram-Schmidt of gas products from thermal decomposition of 1-nitroso-2-naphthol + 5%NaOH.

After adding sodium hydroxide, the dynamic three-dimensional infrared spectrum and gram Schmidt information of the gas product decomposed by 1-nitroso-2-naphthol during heating are shown in Figs. 9 and 10, respectively.

It can be seen from Fig. 9 that after the addition of sodium hydroxide, the infrared peak of the decomposition gas product of 1-nitroso-2 naphthol has strong absorption near 1100 cm^−1^ and 1380 cm^−1^, especially near 1380 cm^−1^. According to the comparison of the spectra, 1380 cm^−1^ is the symmetrical stretching absorption peak of aliphatic nitro compounds, and 1100 cm^−1^ is the antisymmetric stretching vibration peak of ether C–O–C groups.

Compared with Fig. 6, after the addition of sodium hydroxide, the decomposition gas product of 1-nitroso-2 naphthol has a stronger absorption band near 1380 cm^−1^ in addition to the strong absorption of 1100 cm^−1^, indicating that the main reaction has changed after the addition of sodium hydroxide.

**Figure 11:**
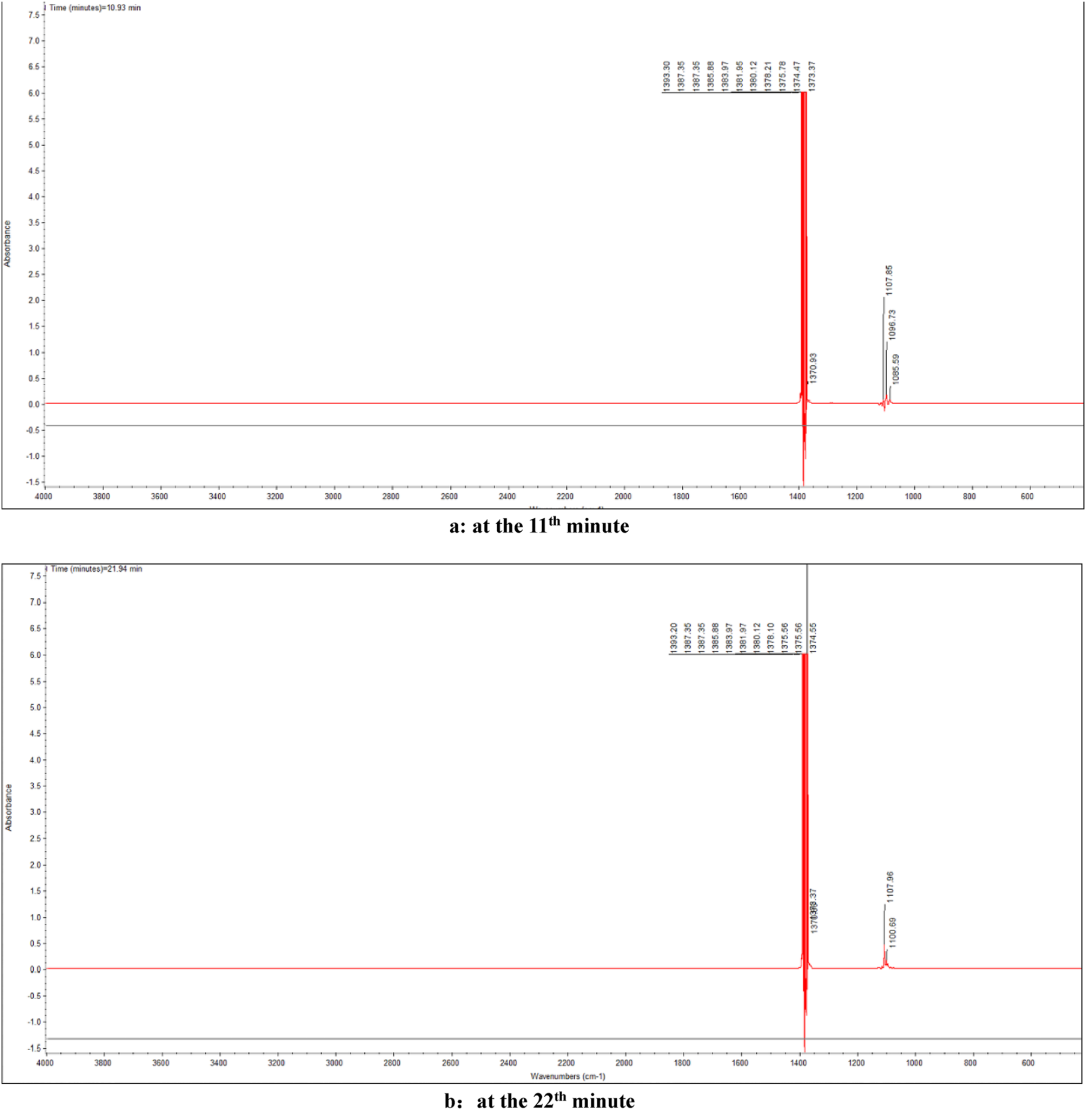
FTIR of gas products from thermal decomposition of 1-nitroso-2-naphthol + NaOH at the 11th minute and the 22th minute.

It can be seen from Fig. 10 that the strong and dense absorption is at about the 11^th^ minute and the 22^th^ minute. Therefore, the infrared absorption spectrum at the time is listed and analyzed separately, as shown in Fig. 11.

Similarly, as can be seen in Fig. 11, after adding sodium hydroxide, the ether bond absorption near 1100 cm^−1^ decreases, the CO_2_ absorption peak near 668 cm^−1^ and 2356 cm^−1^ disappears, and a super strong symmetric stretching absorption peak of aliphatic nitro compounds is added at 1380 cm^−1^. This shows that after adding sodium hydroxide, the dehydration reaction between 1-nitroso-2-naphthol molecules is weakened, more 1-nitroso-2-naphthol reacts with sodium hydroxide to form sodium nitrophenol compounds, and sodium nitrophenol is further heated and decomposed into aliphatic nitro compounds. At the same time, compared with Fig. 8, it is also found that after adding sodium hydroxide, the small peaks in other bands are significantly weakened. Except for the strong absorption peaks near 1100 cm^-1^ and 1380 cm^-1^, the curves of other bands are relatively smooth, indicating that the thermal decomposition path changes after the addition of sodium hydroxide.

## Conclusions

The study results were as follows:

The thermal weight loss of 1-nitroso-2 naphthol has three stages. The first two stages are endothermic process, corresponding to the evaporation of water. The third stage is exothermic decomposition process, and the corresponding maximum exothermic temperature (*T*_p_) is between 144.57 and 172.33 °C.

The heating rate (β) has an effect on the thermal decomposition process of 1-nitroso-2 naphthol. The faster the heating rate (β), the higher the starting temperature (*T*_onset_) and maximum temperature (*T*_p_) of thermal decomposition, the faster the thermal decomposition rate, the greater the heat release and the worse the thermal stability.

After doping a small amount of sodium hydroxide, the initial temperature (*T*_onset_) and the maximum temperature (*T*_p_) of thermal decomposition of 1-nitroso-2 naphthol decreased, and the thermal stability became worse.

The dynamic infrared absorption spectrum of TGA/DSC-FTIR showed that the main reaction of 1-nitroso-2 naphthol during heating was intermolecular dehydration to form ether. After adding sodium hydroxide, the thermal decomposition process of 1-nitroso-2-naphthol changed, the intermolecular dehydration reaction weakened, more 1-nitroso-2-naphthol reacted with sodium hydroxide to form sodium nitrophenol compounds, and finally decomposed into aliphatic nitro compounds.

## Data Availability

The datasets used and/or analysed during the current study available from the corresponding author on reasonable request.
